# Molecular forms of butyrylcholinesterase and obesity

**DOI:** 10.1590/S1415-47572010005000072

**Published:** 2010-09-01

**Authors:** Dellyana R. Boberg, Lupe Furtado-Alle, Ricardo L. R. Souza, Eleidi A. Chautard-Freire-Maia

**Affiliations:** Department of Genetics, Federal University of Paraná, Curitiba, PRBrazil

**Keywords:** butyrylcholinesterase, body mass index, obesity, *BCHE* gene SNPs

## Abstract

This study compared obese (N = 134) and unobese (N = 92) male blood donors, regarding the relative intensity (RI) and activity of different molecular forms (G1, G2, G4 and G1-ALB) of butyrylcholinesterase (BChE, EC 3.1.1.8) found in plasma, thereby searching for an association between these variables with obesity and SNPs of exons 1 and 4 of the *BCHE* gene. It was shown that obese and unobese individuals do not differ in the RI of each BChE band, even when classifying the sample into three genotypes of exons 1 and 4 of the *BCHE* gene (*-116GG/539AA, -116GG/539AT*, -*116GA/539AT*). Although the mean BChE activity of each band was significantly higher in obese than in unobese blood donors, the proportions of BChE bands were maintained, even under the metabolic stress associated to obesity, thereby leading to infer that this proportion is somehow regulated, and may therefore be important for BChE functions.

Human butyrylcholinesterase (BChE; EC 3.1.1.8) hydrolyses choline and other esters, this synthesis taking place in the liver, with posterior distribution to several parts of the organism. BChE is coded by the *BCHE* gene (3q26.1-q26.2) which presents four exons (Arpagaus *et al.*, 1990), with more than 70 already-described variants (review in Souza *et al.*, 2005b). Another type of genetic variation occurs through the interaction of products from the *BCHE* and *CHE2* (2q33-q35) genes, this resulting in the CHE2 C5+ and CHE2 C5- phenotypes, characterized by the presence and absence of the C_5_ complex, respectively (Harris *et al.*, 1962).

BChE is found in human plasma, either in homomeric*,* viz., monomers (G1), dimers (G2), trimers (G3) and tetramers (G4) (Harris *et al.*, 1962), or heteromeric forms associated with other substances, such as albumin (G1-ALB) (Masson, 1989).

Although the physiological role of BChE has not been fully elucidated, published data suggest its relationship with lipid metabolism (Kutty *et al.*, 1977), weight (Chautard-Freire-Maia *et al.*, 1991; Li *et al.*, 2008) and body mass index (BMI) (Alcântara *et al.*, 2001, 2003; Souza *et al.*, 2005a; Furtado-Alle *et al.*, 2008). Besides mean BChE activity tending to be higher in obese than in unobese individuals (Chautard-Freire-Maia *et al.*, 1991; Alcântara *et al.*, 2003; Furtado-Alle *et al.*, 2008), BChE itself is also related to ghrelin (Kojima *et al.*, 2001), a peptide associated with obesity (Tschöp *et al.*, 2001), through its hydrolyzing and inactivating the latter (De Vriese *et al.*, 2004).

The aim was to compare the relative intensity (RI) and activity of BChE bands (G1, G1-ALB, G2 and G4) in obese and control individuals, and search for an association between the activity of each band with obesity and SNPs of exons 1 and 4 of the *BCHE* gene. This research was approved by the Ethical Committee (CONEP; registration number 2063).

The sample comprised 134 obese (BMI ≥ 30; mean age 36.9) and 92 unobese (20 ≤ BMI < 25; mean age 35.1) male blood donors bearing the CHE2 C5- phenotype. Individuals with any no detectable BChE band were excluded from the study. The detection of BChE bands in plasma - stored from eight to eleven years at -20 °C - was by means of electrophoresis (8 h; 100V and 11 mA; 4 °C) of plasma samples (5 μL) mixed with 7 μL of bromophenol blue before loading onto polyacrylamide gels (170 x 160 x 0.8 mm; 29:1 stock solution) of 5% (1 cm; stacking gel) and 7% (13 cm; resolving gel) concentrations in TBE 1X. The bands were revealed at 37 °C after 30 to 40 min incubation in a solution of 0.2% Fast Red TR in 200 mM sodium phosphate buffer (pH 7.1) containing 3.4% of 30 mM alpha-naphthyl acetate dissolved in acetone, the volume being completed with distilled water (1:1). A pre-run of 30 min was done before plasma loading. Data concerning plasma BChE activity (Dietz *et al.*, 1972), as modified (Evans and Wroe, 1978), and genotypes of exons 1 (*-116GG*, *-116GA*, *-116AA*) and 4 (*539AA*, *539AT*, *539TT*) of the *BCHE* gene were obtained from the data bank of the Laboratory of Polymorphisms and Linkage (Genetics Department, UFPR), most of which part of a previous study (Furtado-Alle *et al.*, 2008). The activity of each band was a result of the multiplication of plasma BChE activity by the RI of each BChE band detected in the polyacrylamide gel, subsequently measured by optical densitometry with *KODAK 1D Image Analysis Software.* Statistica for Windows (StatSoft, Inc., 5.5 version, 2000) was used for statistical analysis.

Mean BChE activity in plasma, as estimated for the present sample, was significantly higher in obese (6.51 ± 2.87) than in unobese men (4.61 ± 1.11; t = 6,949, p = 6 x 10^-11^). Table 1 shows that obese and control individuals do not differ in the proportions of each BChE band, although the activity of each band is significantly higher in obese than in unobese men, with ratios varying from 1.32 (G1-ALB) to 1.63 (G1). When classified according to the more frequent genotypes, mean BChE activity in plasma is significantly higher in obese than unobese individuals, respectively: *-116GG/539AA* (N = 80, 6.58 ± 2.66 and N = 54, 5.02 ± 1.10; t = 4.67, p < 10^-5^), *-116GG/539AT* (N = 20, 7.08 ± 3.91 and N = 12, 3.96 ± 1.05; t = 3.37, p < 0.005) and -*116GA/539AT* (N = 19, 5.22 ± 2.22 and N = 12, 3.95 ± 0.78; t = 2.26, p < 0.05). As to RI means of BChE bands, classified according to these genotypes, no significant difference between obese and control individuals was revealed through t-tests (data not shown).

The *K* mutation (*539T*) of the *BCHE* gene was associated with lower BChE activity (Rubinstein *et al.*, 1978), and it was shown that the *-116A* SNP is preferentially found in *cis* combination with the *539T* variant (Bartels *et al.*, 1990). Recently, it was reported that the *539T* SNP alone is not associated with decreased BChE activity, since this requires the 5' UTR *-116A* variant, probably through the latter affecting transcription and/or translation of the *BCHE* gene (Furtado-Alle *et al.*, 2008). From the present data, it can be seen that obese and control samples, grouped by genotypes of exons 1 and 4 of the *BCHE* gene, revealed similar RI of each BChE band. Therefore, the reduced plasma BChE activity associated with genotype *-116GA/539AT* (Furtado-Alle *et al.*, 2008) does not seem to affect the RI of the BChE molecular forms examined.

On considering the encountered similarity of RI in each BChE band in obese and control subjects, the higher mean activity of each band in the former can be attributed solely to higher BChE plasma activity. Even though this activity is higher in obese individuals and lower in the *-116GA/539AT* genotype than in others, from the data, it can be seen that the RI of each band is maintained, independently of obesity and the examined genotypes, suggesting that this proportion is regulated, and may therefore be important for BChE function(s).

## Figures and Tables

**Table 1:** Means ± S. D. of relative intensity and activity of butyrylcholinesterase (BChE) bands in obese (N = 134) and unobese (N = 92) male blood donors, showing the results of t-test comparisons.

BChE bands	Relative intensity				Activity (KU/L)			
	Means ± S.D.		t-test (p)		Means ± S.D.		t-test (p)	Means ratio (O/UO)
	O	UO			O	UO		
G4	0.68 ± 0.13	0.70 ± 0.12	0.96 (> 0.30)		4.39 ± 1.98	3.20 ± 0.86	6.18 (< 10^-8^)	1.37
G2	0.04 ± 0.03	0.04 ± 0.03	0.11 (> 0.90)		0.27 ± 0.23	0.19 ± 0.14	3.37 (< 10^-3^)	1.42
G1-ALB	0.09 ± 0.06	0.09 ± 0.06	0.79 (> 0.40)		0.58 ± 0.43	0.44 ± 0.34	2.75 (< 10^-2^)	1.32
G1	0.19 ± 0.10	0.17 ± 0.09	1.72 (> 0.05)		1.27 ± 1.06	0.78 ± 0.50	4.65 (< 10^-5^)	1.63

S.D. = Standard Deviation, O = Obese sample, UO = Unobese sample.
